# Warming enhances the detrimental impacts of drought and salinity on maize growth, water-use efficiency, and nitrogen recovery

**DOI:** 10.3389/fpls.2026.1764828

**Published:** 2026-05-01

**Authors:** Giacomo Gargano, Gaetano Amato, Calogero Frangiamore, Rosolino Ingraffia, Antonella Lo Porto, Paolo Ruisi, Dario Giambalvo

**Affiliations:** Department of Agricultural, Food and Forest Sciences, University of Palermo, Palermo, Italy

**Keywords:** abiotic stress, multifactorial stress combination, non-additive responses, NUE, WUE

## Abstract

**Introduction:**

Climate change is increasing the co-occurrence of heat, drought, and soil salinization in Mediterranean agroecosystems, yet their interactive effects on crop physiology remain poorly understood. We investigated how warming modifies maize growth and resource-use efficiency when combined with drought or salinity during early vegetative development.

**Methods:**

A pot experiment was conducted in a semi-controlled wire house. Three air temperature regimes were established: ambient conditions and two warming treatments that resulted in mean temperature increases of +1.1 °C and +1.9 °C relative to ambient, despite target increases of +1.5 °C and +4.0 °C. In addition, three water regimes were imposed: well-watered, water stress, and salt stress. Plant growth, water- and N-use efficiency, ^15^N fertilizer recovery, and Δ^13^C discrimination were measured. Additivity of combined stress effects was evaluated using bootstrap-based effect size analyses.

**Results:**

Salinity imposed the strongest individual constraints on growth, water consumption, and N uptake, while drought induced moderate shoot reduction but enhanced root growth. Warming alone had limited effects on biomass but increased water demand. Under combined stress, warming amplified the detrimental impacts of both drought and salinity on plant performance, sometimes resulting in non-additive responses, including synergistic declines in water-use efficiency and N recovery.

**Discussion:**

These findings show that maize responses to multifactorial stress cannot be predicted from single-stress behaviors. Furthermore, the results showed a progressive intensification of the negative effects of combined stresses on plant performance with increasing temperature. Therefore, considering that the temperature increases effectively achieved were substantially lower than those projected under the most severe climate change scenarios, it follows that, if such projected temperature increases were fully realized, the resulting negative impacts would be even more pronounced.

## 1 Introduction

Global climate change is driving increasingly extreme growing conditions in many crop regions, especially in Mediterranean and semi‐arid zones (Zittis et al., 2022). Increasing evidence from climate models and observational studies highlights a significant upward trend in the frequency and severity of extreme heat and drought events worldwide (IPCC, 2023; Richardson et al., 2023). In the Mediterranean basin, temperatures are rising faster than the global average threatening crop yields and agricultural systems resilience (Zhao et al., 2017; Zittis et al., 2022; IPCC, 2023). At the same time, reduced precipitation and higher evapotranspiration rates exacerbate salt accumulation in the root zone, leading to increased soil salinization, an already critical issue in many dryland agricultural systems (IPCC, 2023). Global assessments find that soil salinization is expected to worsen with climate change (Hassani et al., 2021).

Drought, heat, and salinity each impair plant function through distinct physiological mechanisms. Drought conditions cause soil moisture deficits that directly limit plant water uptake, reducing leaf turgor pressure and stomatal conductance, and consequently impairing photosynthetic capacity and growth (Mukarram et al., 2021). Salinity often accompanies drought, particularly in arid and semi-arid agricultural systems, compounding plant water deficits through osmotic effects and inducing ionic toxicity from excess sodium (Na^+^) and chloride (Cl^-^) ions. This leads to disrupted nutrient uptake, imbalance of essential nutrients such as potassium and calcium, and elevated oxidative stress (Munns and Tester, 2008; Zandalinas et al., 2020). Heat stress further complicates these interactions by accelerating plant phenology, increasing evaporative demand, and causing elevated transpiration rates, potentially leading to reduced biomass accumulation and nutrient efficiency, even in vegetative growth stages (Moore et al., 2021).

In natural field conditions, crops rarely experience these stresses in isolation. Agricultural environments are inherently complex, with multiple abiotic factors fluctuating simultaneously and interacting dynamically over time. Although significant research has focused on individual stressors—such as drought, heat, or salinity—relatively few studies have systematically investigated plant responses to combinations of these stresses, particularly under realistic environmental scenarios. This gap is especially evident during the early vegetative growth stages, which are critical for determining plant establishment, resource allocation, and ultimately potential productivity. Recent evidence suggests that plant responses to multiple concurrent stresses are often non-additive, with synergistic or antagonistic interactions that can amplify physiological disruption, impair photosynthetic efficiency, and accelerate oxidative damage beyond what would be predicted from single-stress analyses (Rillig et al., 2019; Zandalinas and Mittler, 2022; Kamatchi et al., 2024). Such complex interactions can alter hormonal signaling pathways, metabolic adjustments, and gene expression networks (Cohen et al., 2021), challenging our ability to predict plant performance under future climate scenarios characterized by increasing stress co-occurrence. Despite these advances, the current availability of detailed experimental data addressing combined stress interactions remains limited, underscoring the need for integrative approaches that couple agronomic and physiological, and biochemical analyses to better understand the mechanisms underlying plant response to multifactorial stress environments.

This study aims to address this critical research gap by thoroughly examining maize (*Zea mays* L.) physiological and growth responses under realistic and agronomically relevant combinations of temperature increments and either water deficit or soil salinity—stressors whose frequency and severity are projected to increase in Mediterranean agroecosystems due to ongoing climate change. The experiment was conducted under environmental conditions in which air temperatures during the vegetative phase of maize frequently exceed 30 °C; under such conditions, even small temperature increases may further exacerbate the stress levels inherently experienced by plants. Hence, the objectives of this work were to: i) quantify the effects of individual and combined stress conditions (warming × water deficit; warming × salinity) on maize growth (both shoot and root biomasses); ii) assess plant water and nitrogen (N) use efficiencies in order to determine how maize adjusts water economy and N uptake when exposed to multiple concurrent stressors; iii) identify potential additive or non-additive (synergistic or antagonistic) interactions between warming and the two abiotic stress factors (water deficit and salinity), with the aim of determining whether combined stress responses can be predicted from single-stress behaviors or represent fundamentally distinct physiological outcomes.

## 2 Materials and methods

### 2.1 Experimental site and setup

The experiment was conducted in pots at the Pietranera farm (Lima Mancuso Foundation, Santo Stefano Quisquina, AG, Italy; 37°32’39.54” N, 13°31’01.32” E; 162 m a.s.l.) in a wire house covered with a transparent plastic roof with open sides; the wire house shielded the experimental units from natural rain while permitting natural temperature variations.

A split-plot design with ten replicates was adopted, with three temperature regimes as main plots and three water regimes as subplots. The temperature treatments were designed to impose constant increases of +1.5 °C (T1) and +4.0 °C (T2) above ambient conditions (control) throughout the experiment. In practice, however, the achieved mean temperature increases were +1.1 °C and +1.9 °C for T1 and T2, respectively, reflecting deviations from the planned target values. The water regimes included a well-watered control (WW, maintaining soil moisture between 70 and 90% of field capacity), a water-stress treatment (WS, 40–60% of field capacity), and a salt-stress treatment (SS, maintaining soil moisture between 70 and 90% of field capacity using irrigation water with an electrical conductivity of 50 mS cm^-1^, until the soil saturation extract reached an electrical conductivity of approximately 8 mS cm^-1^). Each of the 3 × 3 treatment combinations was replicated ten times, for a total of 90 pots.

**Figure 1:**
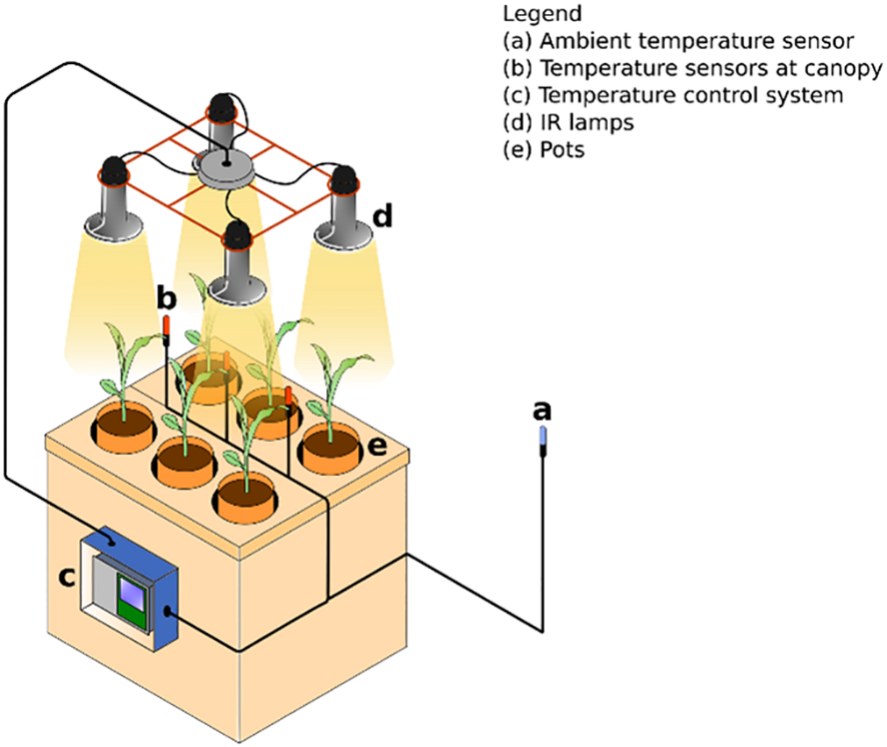
Diagram of the experimental setup built to conduct the study.

Temperature increases were achieved using 100 W ceramic infrared lamps suspended above the canopy on an adjustable frame, with air temperature continuously monitored by temperature sensors. A control system featuring embedded technology was implemented to switch on the lamps when the temperature dropped by more than 0.5 °C below the setpoint and to switch them off once the target temperature was reached. The height of the lamps was adjusted twice per week to maintain a constant distance of approximately 10 cm from the canopy (**Figure 1**).

Pots (2.51 L; 8 cm diameter, 50 cm height) were filled with a 70:30 (w/w) mixture of agricultural soil and washed river sand. The pot sizes were selected to ensure, based on evidence from previous studies on the same species, that the ratio between plant biomass and pot volume remained close to 1 until the conclusion of the experiment. This threshold has been identified by Poorter et al. (2012) as suitable for minimizing the risk of influencing the relative differences among treatments. The agricultural soil (Typic Xerorthent, sieved to <2 mm) had the following properties: 267 g kg^-1^ clay, 247 g kg^-1^ silt, and 486 g kg^-1^ sand; pH 8.0; total C 10.8 g kg^-1^ (Walkley–Black); total N 0.86 g kg^-1^ (Kjeldahl); available P 40.1 mg kg^-1^ (Olsen P); total P 598 mg kg^-1^; cation exchange capacity 26 cmol kg^-1^; saturated electrical conductivity 1.70 dS m^-1^ at 25 °C; water content at field capacity 27.9%; and water content at the permanent wilting point 18.9%. The river sand (Gras Calce Srl, Trezzo sull’Adda, Italy) had a concentration of N (Kjeldahl) and P (Olsen) of 0.11 g kg^−1^ and 7.44 mg kg^−1^, respectively.

Maize (cv. MAS 16.B) was used as the test species. Seeds were sown on 24 May 2024, with three seeds per pot, and thinned to one plant after emergence. Each pot received 190 mg of ammonium sulfate (equivalent to 80 kg N ha^-1^) with a 10% enrichment of ^15^N isotope, applied in two equal doses at 8 and 13 days after emergence. Soil water content was monitored gravimetrically three times per week, and water was added whenever soil moisture reached the predetermined thresholds of water-holding capacity (70% or 40%). Water stress treatments were imposed seven days after plant emergence.

At 30 days after emergence (corresponding to the V3–V4 vegetative stage), the shoot biomass from each pot was harvested and oven-dried at 40 °C to a constant weight to determine dry matter. Root biomass was carefully extracted by sieving and washing, then oven-dried at 40 °C to a constant weight. Subsequently, both shoot and root fractions were ground to a fine powder using a Qiagen TissueLyser II. Subsamples representing 30% of the total shoot and 30% of the total root dry weight were combined to form a composite sample, which was analyzed for N concentration and for ^15^N and ^13^C abundance using an elemental analyzer (NA1500; Carlo Erba, Milan, Italy) coupled to an isotope ratio mass spectrometer (Isoprime; Cheadle, UK).

Total N uptake was calculated by multiplying the N concentration of the biomass by the corresponding biomass dry weight in each pot. The ^15^N enrichment was used to determine the amount (^15^Nrec) and percentage (%^15^Nrec) of fertilizer-derived N, according to the following equations:


Nrec15=Nt×atom%15Np excessatom%15Nf excess

And


%15Nrec=100×Nrec15f

where N_t_ is N content (g pot^−1^) in the biomass, atom% ^15^Np excess is the ^15^N isotopic excess (atom% ^15^N − 0.3663) in the fertilized plant, atom% ^15^Nf is the ^15^N isotopic excess in the fertilizer, and f is the amount of fertilizer (g pot^−1^).

The δ^13^C content was expressed in δ^13^C‰ units using the international PDB (Pee Dee Belemnite) standard, according to the following equation:


$${\text{δ}}^{13}\text{C}=(\frac{\text{Rsample}}{\text{Rstandard}}-1)\times 1000$$

The δ^13^C is a relative value where the C isotope ratio ^13^C/^12^C (R) of the sample is compared with the ratio of the international PDB standard. The δ^13^C content of the plant material is related to ^13^C isotope discrimination (Δ) by the following equation:


$$\text{Δ}=\frac{\left({\text{δ}}^{13}\text{Cair}-{\text{δ}}^{13}\text{Cplant}\right)}{\left(1+\frac{{\text{δ}}^{13}\text{Cplant}}{1000}\right)}$$

where δ^13^Cair is the δ^13^C value of air (–8.15‰) and δ^13^Cplant is the measured value of the plant material.

Data obtained from each experimental unit were used to determine the N use efficiency (NUE), according to the following equation:


$$\text{NUE}=\frac{\text{biomass production}}{{\text{N}}_{\text{t}}}$$

Finally, water use efficiency (WUE) was calculated according to the following equation:


$$\text{WUE}=\frac{\text{biomass production}}{\text{water consumption}}$$

where water consumption represents the total amount of water applied throughout the experiment.

### 2.2 Statistical analysis

All statistical analyses were conducted in R version 4.3.2 (R Core Team, 2024). A two-way ANOVA following a split-plot design (Montgomery, 2017) was used to assess the effects of temperature regime (main-plot) and water regime (sub-plot), both treated as fixed effects with ten replicates. Data were analysed with “aov()” function, reflecting the hierarchical layout of the experimental design via nested error terms. Model residuals were checked for normality and homogeneity of variance using Shapiro–Wilk test and Levene’s test, respectively. When assumptions were violated, appropriate transformations were applied prior to analysis.

To evaluate the effects of our treatments compared to the control treatment (WW), a non-parametric bootstrap approach using the “dabestr” package (Ho et al., 2019) based on 5,000 iterations (resampling with replacement) was implemented. Effect sizes were calculated as unpaired mean differences (Δ-mean) between treatment and control group, with bias-corrected and accelerated bootstrapped 95% confidence intervals (CIs). For the single-stress treatments (T1, T2, WS, and SS) comparisons were set against the untreated control (WW); for the combined-stress treatments [WS(T1) and WS(T2), and SS(T1) and SS(T2)] comparisons were set against their respective single-stress baselines WS and SS, respectively. We used this combined approach given the increasing recognition of the limitations of using only *P* values and avoiding dichotomous cutoffs (Ho et al., 2019; Wasserstein and Lazar, 2020).

To evaluate whether the combined effects of two concurrent stressors were additive, a bootstrap-based additivity index for each double-stress treatment was computed. For a given combination, the index was calculated as:


$$\text{Additivity Index }={\text{ES}}_{\text{ij}}\;-\;({\text{ES}}_{\text{i}}\;+{\text{ ES}}_{\text{j}})$$

where ES_ij_ is the effect size of the double-stress treatment *vs.* the untreated control (WW), ES_i_ and ES_j_ are the effect sizes of the corresponding single-stress treatments *vs.* the same control. An interaction was deemed additive if the 95% CIs of the index included zero; otherwise, it was classified as non-additive.

To assess overall treatment differentiation across multiple variables, we conducted a Canonical Discriminant Analysis (CDA) on the nine measured response variables. The analysis was implemented using the “candisc” package (Friendly and Fox, 2025). More details on statistical methods adopted to perform CDA may be found in Supplementary material.

Graphical representations were generated using the “tidyverse” meta-package (Wickham et al., 2019) which includes the package ggplot2 (Wickham, 2016).

## 3 Results

### 3.1 Climatic parameters

**Figure 2:**
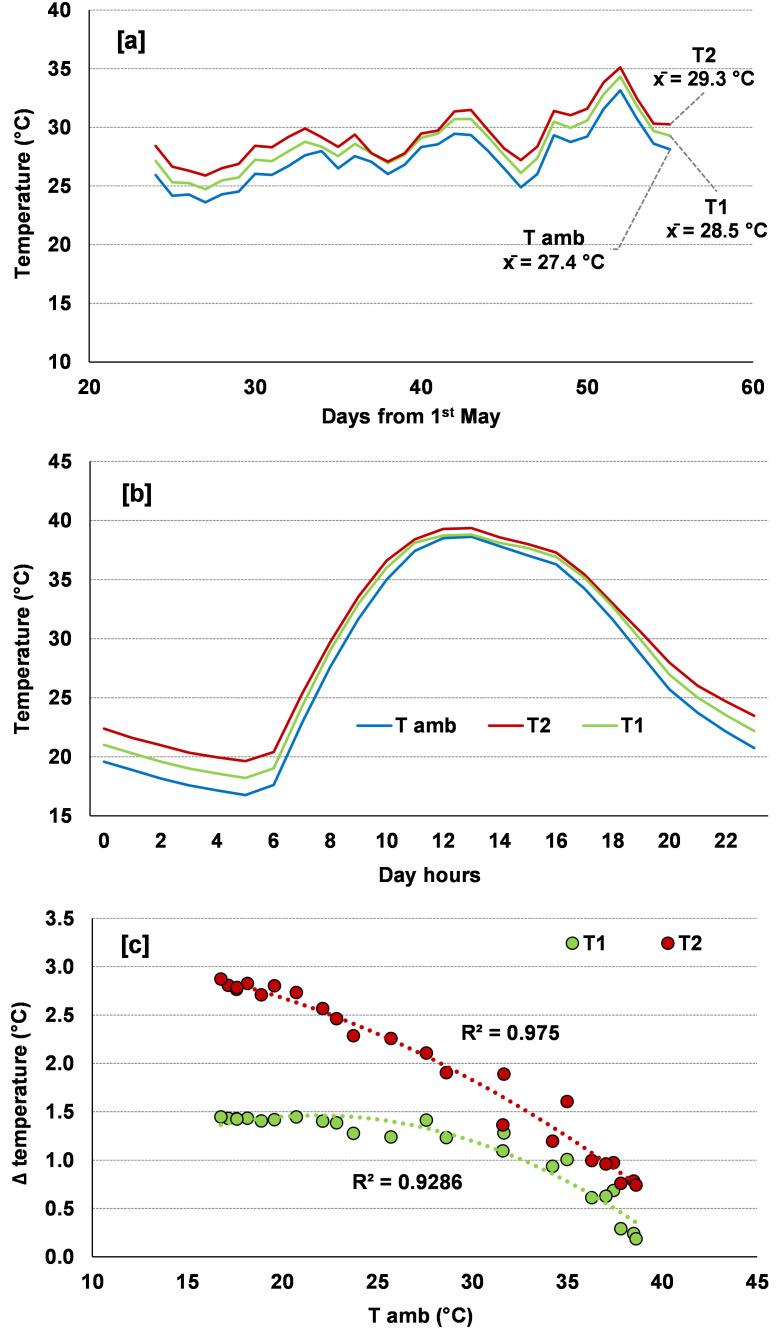
Mean temperature during the experimental period for each temperature treatment **(A)** (T amb: ambient temperature; T1 and T2: scheduled temperatures of +1.5 and +4.0 °C above ambient temperature). Diurnal temperature patterns for each temperature treatment **(B)**. Relationships between the variation (Δ) in the temperatures induced by the infrared lamps in T1 and T2 and the ambient temperature **(C)**.

The mean daily ambient temperatures during the experimental period ranged from 23 °C to 33 °C, with an average slightly above 27 °C (**Figure 2A**). The infrared lamps increased on average the temperatures by 1.1 °C in the T1 treatment and by 1.9 °C in the T2 treatment, which are valued considerably lower than the programmed targets. Analysis of the diurnal temperature patterns clearly shows that the infrared lamps were most effective during nighttime hours, approaching the target thresholds, and their effect progressively decreased as ambient temperatures rose during the day (**Figure 2B**). Consequently, a clear relationship emerged between lamp-induced temperature increases and ambient temperature (**Figure 2C**). Specifically, in the T1 treatment, lamp effectiveness was optimal when ambient temperatures remained below 25 °C, then gradually declined as temperatures increased. In the T2 treatment, the programmed target was never reached; at ambient temperatures below 20 °C, lamp effectiveness was approximately 75% (resulting in increases of about 3 °C compared to the +4 °C target), and it decreased almost linearly with increasing temperatures, so that when temperatures exceeded 35 °C, the observed increases were only slightly above 1 °C.

### 3.2 Plant growth and resource use efficiency

Results of the ANOVA and of the contrasts to evaluate the effects of stress treatments compared to the control treatments are reported in **Supplementary Table 1**; **Table 1**, respectively.

**Table 1:** The Δ-mean and the confidence intervals (in parentheses) for selected treatment contrasts relative to the relative control treatment [(T1, T2, WS, and SS *vs.* WW; WS(T1) and WS(T2) *vs.* WS; and SS(T1) and SS(T2)] *vs.* SS.

Trait	Statistic parameter	*vs.* WW				*vs.* WS		*vs.* SS	
		T1	T2	WS	SS	WS(T1)	WS(T2)	SS(T1)	SS(T2)
Shoot	Delta mean	-0.067	-0.058	-0.248	-0.612	-0.052	-0.138	-0.048	-0.065
	*CIs 95%*	*[-0.19; 0.06]*	*[-0.21; 0.09]*	*[-0.36; -0.14]*	*[-0.75; -0.47]*	*[-0.19; 0.08]*	*[-0.26; -0.01]*	*[-0.22; 0.13]*	*[-0.23; 0.10]*
Root	Delta mean	-0.003	-0.057	0.071	-0.371	-0.029	-0.064	-0.059	0.067
	*CIs 95%*	*[-0.09; 0.09]*	*[-0.15; 0.03]*	*[-0.02; 0.16]*	*[-0.45; -0.29]*	*[-0.11; 0.05]*	*[-0.18; 0.05]*	*[-0.13, 0.01]*	*[-0.01; 0.14]*
Water consumption	Delta mean	0.144	0.233	-0.237	-0.687	0.063	0.151	0.051	0.188
	*CIs 95%*	*[0.05; 0.24]*	*[0.14; 0.33]*	*[-0.31; -0.17]*	*[-0.79; -0.59]*	*[0.01; 0.11]*	*[0.09; 0.21]*	*[-0.05; 0.15]*	*[0.10; 0.28]*
WUE	Delta mean	-0.078	-0.143	-0.033	-0.023	-0.073	-0.173	-0.091	-0.199
	*CIs 95%*	*[-0.14; -0.01]*	*[-0.321; -0.08]*	*[-0.09; 0.03]*	*[-0.12; 0.07]*	*[-0.16; 0.00]*	*[-0.24; -0.11]*	*[-0.23; 0.05]*	*[-0.32; -0.07]*
N concentration	Delta mean	-0.001	-0.038	0.009	0.378	0.063	0.011	0.103	-0.047
	*CIs 95%*	*[-0.10; 0.10]*	*[-0.13; 0.05]*	*[-0.10; 0.12]*	*[0.24; 0.50]*	*[-0.05; 0.17]*	*[-0.13; 0.10]*	*[-0.04; 0.25]*	*[-0.18; 0.10]*
N uptake	Delta mean	-1.518	-1.883	-5.628	-11.235	-0.605	-3.352	-0.511	-2.109
	*CIs 95%*	*[-4.59; 1.51]*	*[-5.23; 1.43]*	*[-8.20; -3.00]*	*[-14.75; -7.70]*	*[-5.09; 4.14]*	*[-6.18; -0.67]*	*[-5.09; 4.13]*	*[-6.17; 2.04]*
^15^N fertilizer recovery	Delta mean	1.117	0.649	-3.543	-2.082	-0.313	-0.198	-0.600	-1.389
	*CIs 95%*	*[-0.02; 2.27]*	*[-0.64; 1.90]*	*[-4.46; -2.61]*	*[-3.48; -0.72]*	*[-1.16; 0.55]*	*[-1.05; 0.71]*	*[-2.27; 1.16]*	*[-2.83; 0.03]*
NUE	Delta mean	-0.048	0.651	-0.184	-6.037	-1.129	0.216	-1.396	0.627
	*CIs 95%*	*[-1.90; 1.82]*	*[-1.08; 2.39]*	*[-2.28; 1.92]*	*[-8.15; -3.79]*	*[-3.19; 0.91]*	*[-1.98; 2.46]*	*[-3.44; 0.59]*	*[-1.47; 2.58]*
Δ^13^C	Delta mean	0.034	-0.031	0.182	0.157	0.010	-0.112	-0.151	-0.106
	*CIs 95%*	*[-0.05; 0.12]*	*[-0.11; 0.04]*	*[0.10; 0.26]*	*[0.03; 0.29]*	*[-0.07; 0.09]*	*[-0.20; -0.03]*	*[-0.29; -0.01]*	*[-0.22; 0.00]*

**Figure 3:**
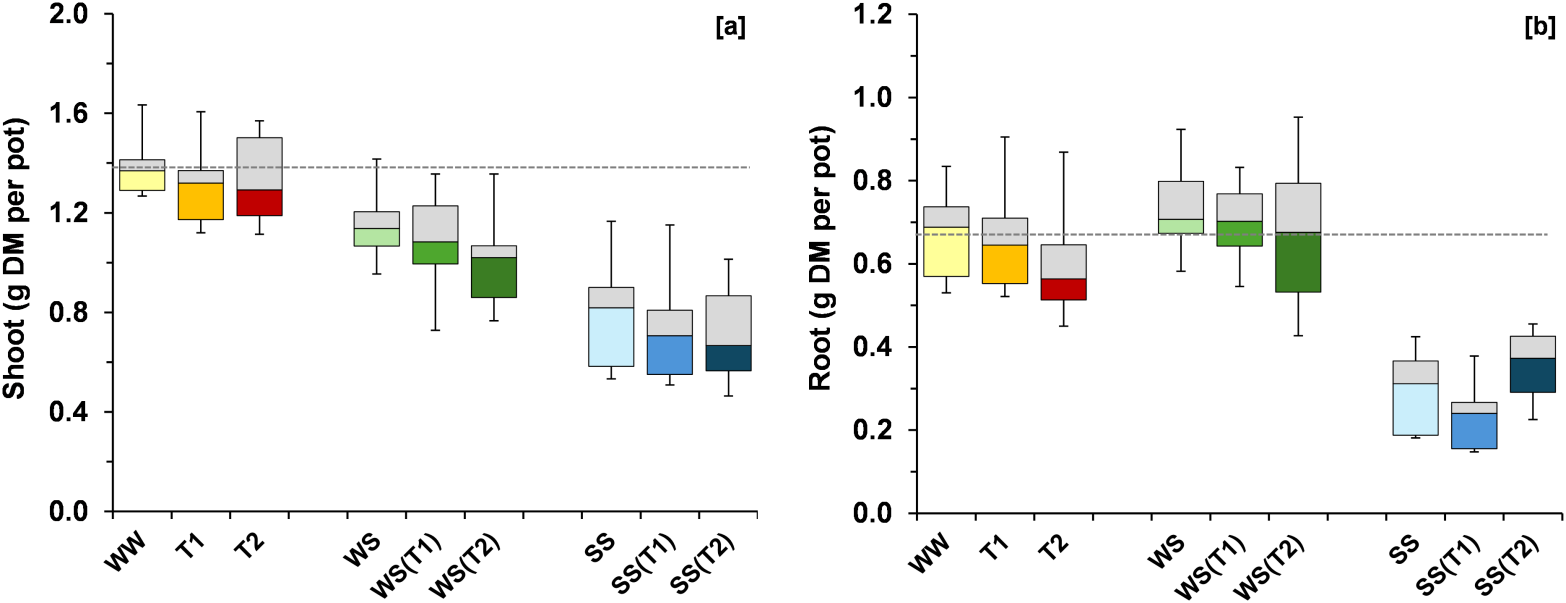
Boxplots showing variation around the median for shoot **(A)** and root **(B)** biomass per pot for each treatment [WW: control, i.e., well-watered condition under ambient temperature; T1 and T2: scheduled temperatures of +1.5 and +4.0 °C above ambient temperature; WS: water stress; WS(T1): water stress + T1; WS(T2): water stress + T2; SS: salt stress; SS(T1): salt stress + T1; SS(T2): salt stress + T2]. The grey dashed line was drawn at the mean value of the control treatment (WW).

The imposition of the T1 regime resulted in modest (non-significant) reductions in both shoot and root biomass (approximately –5% and –1%, respectively; **Figures 3A, B**). A further temperature increase (T2 regime) did not substantially alter this pattern, although the reduction in root biomass appeared more pronounced, amounting to an 8% decrease compared to the control (WW). Under ambient temperature, water stress (WS) reduced shoot biomass by approximately 20% relative to WW, whereas root biomass showed a slight increase (~9%). Salt stress (SS) induced the most pronounced decreases in both shoot and root biomass (–45% and –54%, respectively). Increases in temperature led to additional decline in plant growth. Water stress decreased shoot biomass by a further 5% at T1 and 12% at T2. Under SS, root biomass declined by 20% at T1; however, unexpectedly, it increased by 23% at T2.

**Figure 4:**
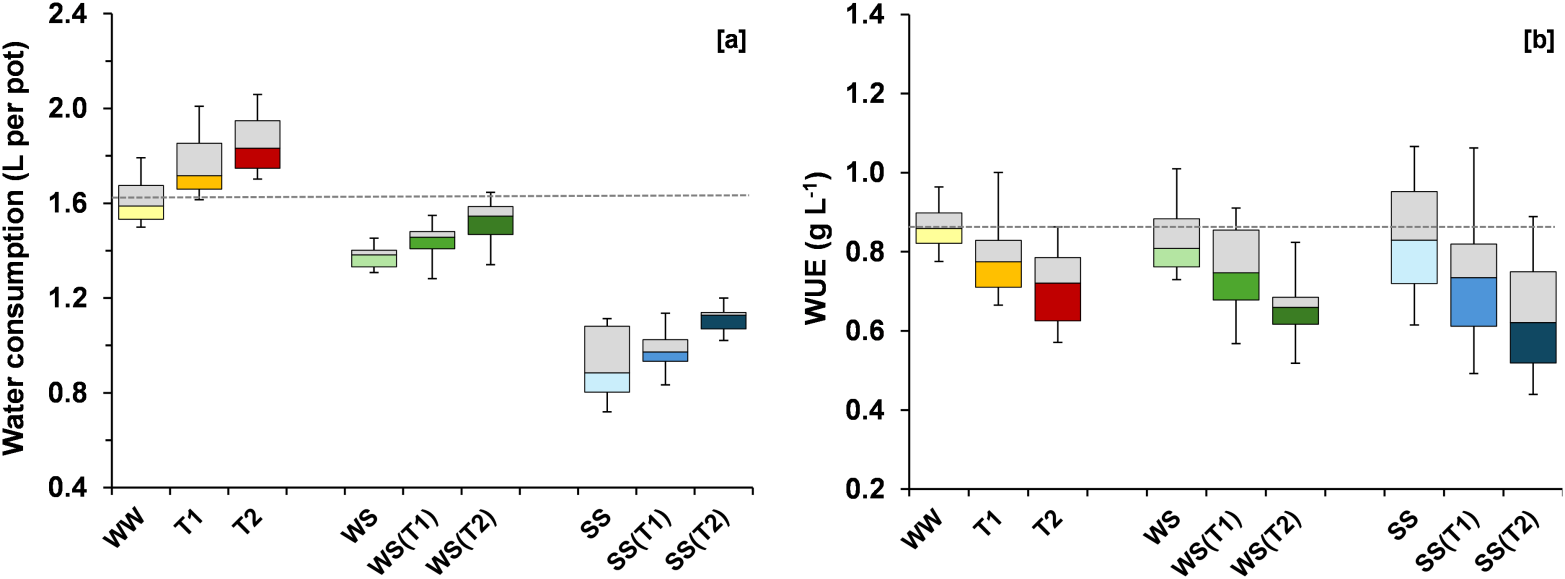
Boxplots showing variation around the median for water consumption **(A)** and water use efficiency (WUE) **(B)** for each treatment [WW: control, i.e., well-watered condition under ambient temperature; T1 and T2: scheduled temperatures of +1.5 and +4.0 °C above ambient temperature; WS: water stress; WS(T1): water stress + T1; WS(T2): water stress + T2; SS: salt stress; SS(T1): salt stress + T1; SS(T2): salt stress + T2]. The grey dashed line was drawn at the mean value of the control treatment (WW).

Salt stress reduced water consumption by 42% relative to WW conditions, while WS led to a 17% reduction (**Figure 4A**). Increasing temperature intensified water consumption in all treatments, with raises of 20%, 11%, and 15% in SS(T2), WS(T2), and WW(T2), respectively, compared to the control WW. Water use efficiency (WUE) showed a modest (non-significant) decrease under stress conditions, by 6% in SS and 5% in WS compared to the control WW (**Figure 4B**). Increasing temperature induced a progressive and pronounced reduction in WUE in all treatments (WW, WS, and SS).

**Figure 5:**
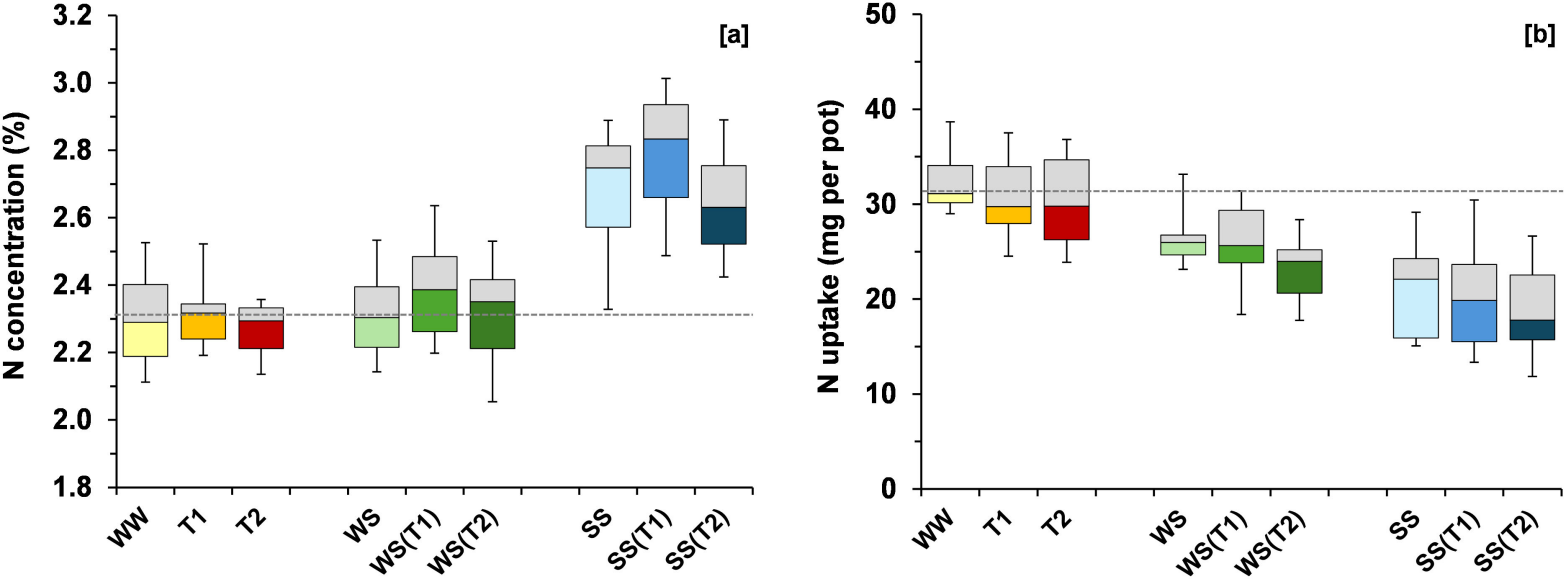
Boxplots showing variation around the median for plant N concentration **(A)** and N uptake **(B)** for each treatment [WW: control, i.e., well-watered condition under ambient temperature; T1 and T2: scheduled temperatures of +1.5 and +4.0 °C above ambient temperature; WS: water stress; WS(T1): water stress + T1; WS(T2): water stress + T2; SS: salt stress; SS(T1): salt stress + T1; SS(T2): salt stress + T2]. The grey dashed line was drawn at the mean value of the control treatment (WW).

Salt stress increased shoot N concentration by 16% relative to WW, while WS did not significantly affect it (**Figure 5A**). Under increasing temperature, N concentration under SS conditions increased slightly at T1 (+3.8%) but declined modestly at T2 (–1.7%). Under WS, N concentration remained stable, whereas only minor variations were observed under WW conditions. Total N uptake was markedly reduced under SS and WS (–35% and –17%, respectively; **Figure 5B**) relative to WW. Under SS, N uptake exhibited an additional 10% decline at T2, while under WS the temperature-induced reduction reached 13%. In the WW treatment, the imposition of the T2 regime caused a moderate decrease of about 6%.

**Figure 6:**
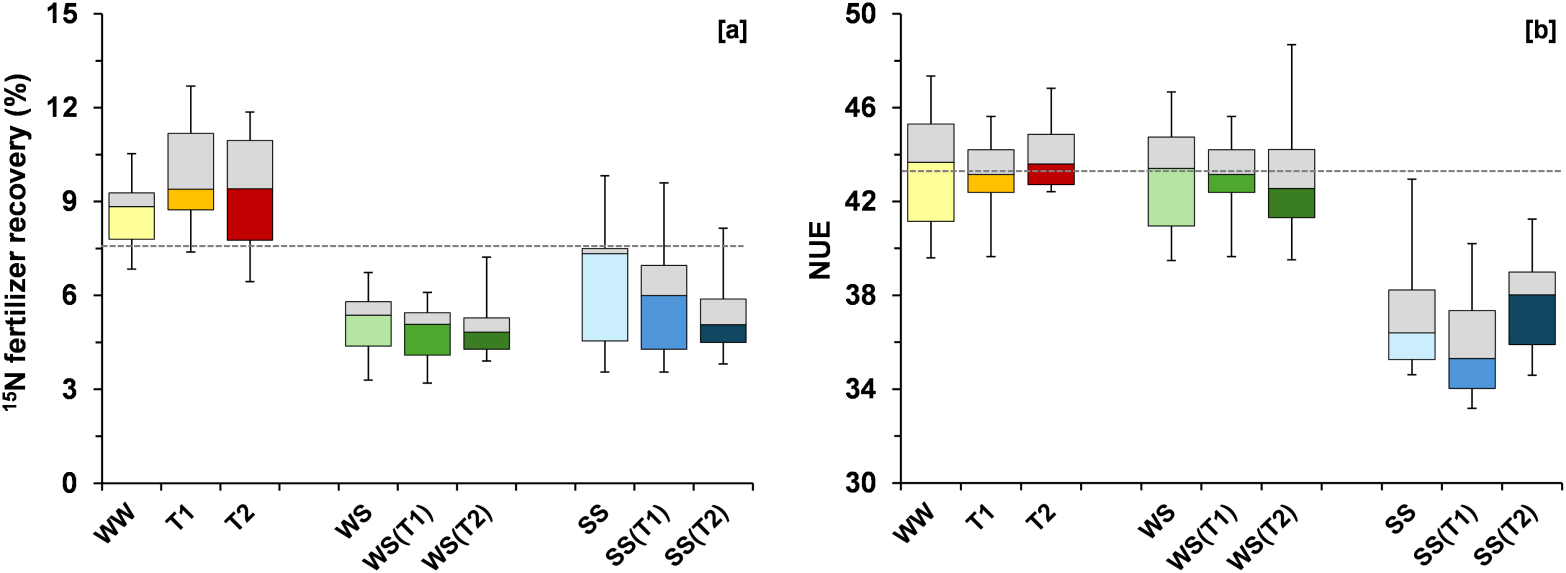
Boxplots showing variation around the median for ^15^N fertilizer recovery **(A)** and N use efficiency (NUE) **(B)** for each treatment [WW: control, i.e., well-watered condition under ambient temperature; T1 and T2: scheduled temperatures of +1.5 and +4.0 °C above ambient temperature; WS: water stress; WS(T1): water stress + T1; WS(T2): water stress + T2; SS: salt stress; SS(T1): salt stress + T1; SS(T2): salt stress + T2]. The grey dashed line was drawn at the mean value of the control treatment (WW).

The ^15^N fertilizer recovery decreased by 24% under SS and by 40% under WS relative to WW (**Figure 6A**). Increasing temperature exacerbated this reduction under SS (–21% at T2), whereas it enhanced the ^15^N fertilizer recovery in WW by 13% at T1 and 7% at T2. Nitrogen use efficiency (NUE) declined by 14% under SS and remained unchanged under WS (**Figure 6B**). Temperature increases produced only minor effects, and NUE remained relatively stable across temperature treatments within each irrigation condition.

Under WW, increasing temperature (both T1 and T2) induced no changes in the Δ^13^C (**Supplementary Figure S1**), which instead significantly, but slightly, increased under both WS and SS conditions relative to WW (+3.1% and +2.7%, respectively). Under both WS and SS, the effects of the increased temperature were modest, generally leading to a reduction of the Δ^13^C value.

**Figure 7:**
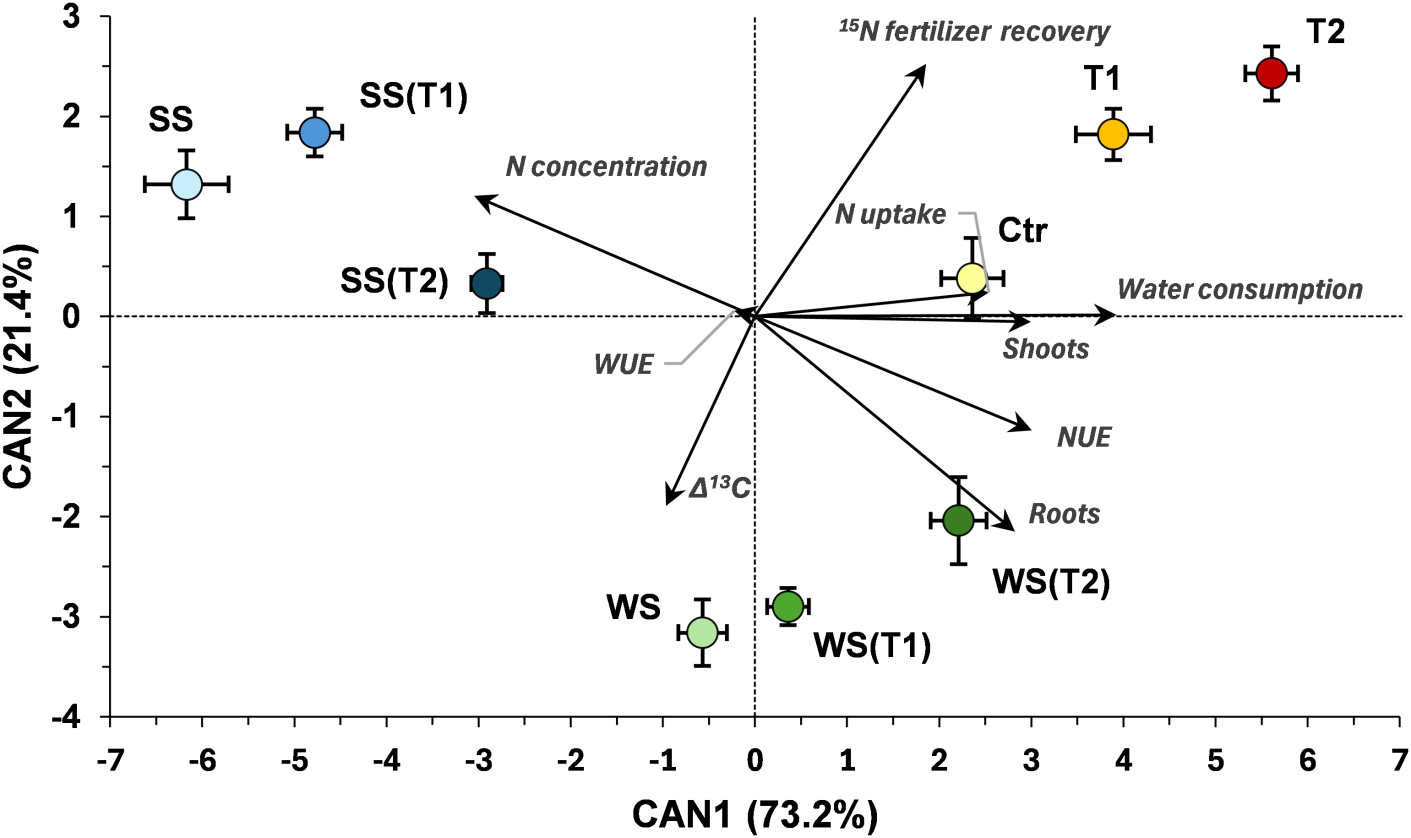
Canonical discriminant analysis (CDA). Canonical variable means (centroid values) were calculated for each treatment [WW: control, i.e., well-watered condition under ambient temperature; T1 and T2: scheduled temperatures of +1.5 and +4.0 °C above ambient temperature; WS: water stress; WS(T1): water stress + T1; WS(T2): water stress + T2; SS: salt stress; SS(T1): salt stress + T1; SS(T2): salt stress + T2]. CAN1, first canonical variable; CAN2, second canonical variable. CDA was performed based on the nine traits measured: shoot and root biomasses, water consumption, water use efficiency (WUE), N concentration, N uptake, ^15^N fertilizer recovery, nitrogen use efficiency (NUE), and Δ^13^C. Within the diagram, the direction and length of each line indicate the canonical loadings of the measured traits on the first two canonical variables.

The CDA performed on data from the nine measured response variables provided a clear separation among the treatments (**Figure 7**). The first canonical variable (CAN1), which explained 73.2% of the variability, effectively separated SS from WW and was mainly influenced positively by water consumption, NUE, and shoot biomass, and negatively by N concentration. The second canonical variable (CAN2) accounted for 21.4% of the variability and distinguished WS from WW, with the most discriminant traits being ^15^N fertilizer recovery (positive loading) and root biomass (negative loading). Mahalanobis squared distances revealed highly significant differences among WW, WS, and SS (*P* < 0.001; Tab. S2). The lower temperature increase (i.e., T1) never produced significant differences within any irrigation regime, whereas Mahalanobis distances markedly increased with the higher temperature (T2), although significance was reached only for WW and SS, not for WS.

**Figure 8:**
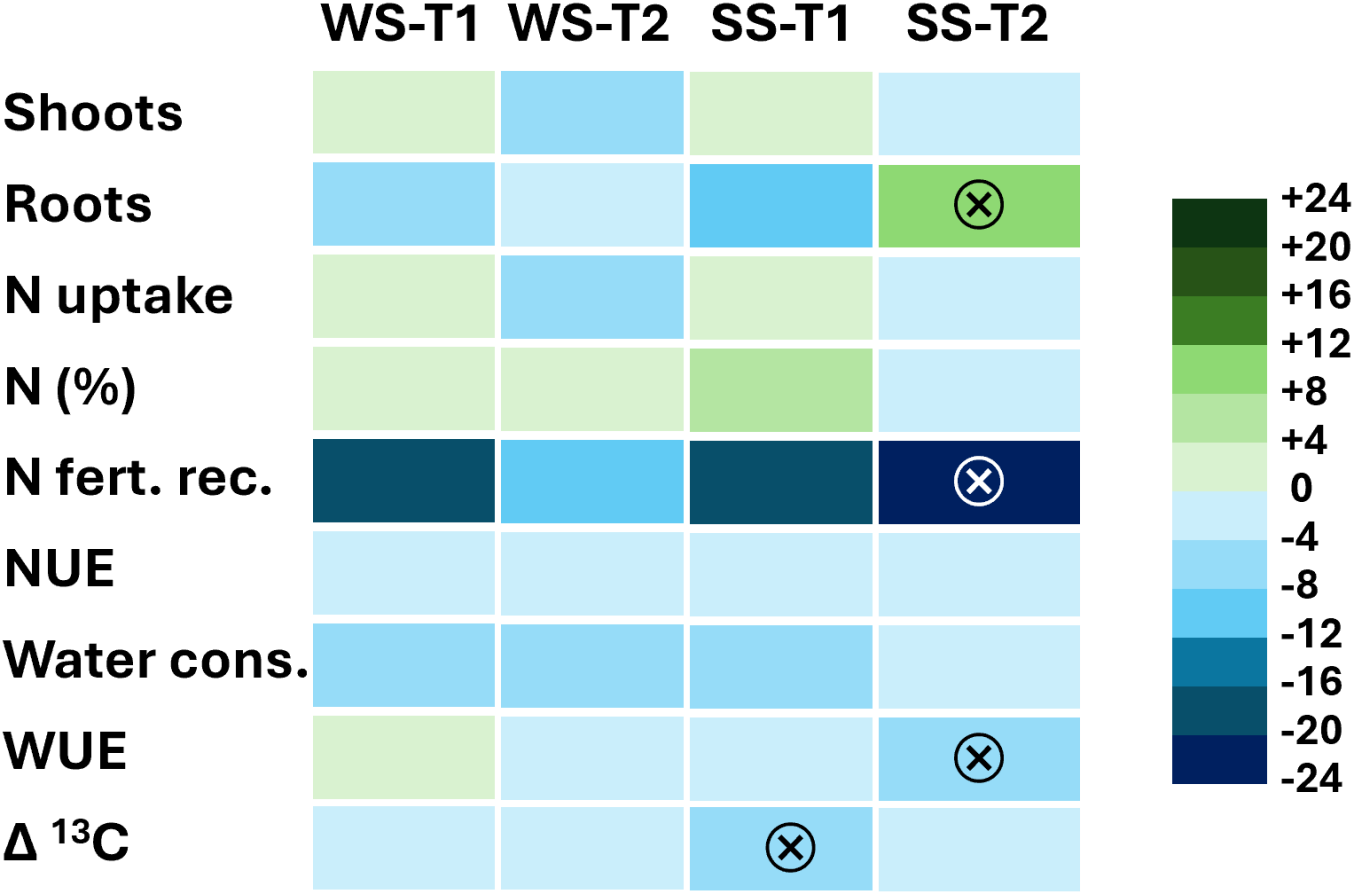
Heatmap of deviations from additive values, calculated as a percentage relative to WW (unstressed control). Values closer to zero (full additivity) are shown in lighter colors, whereas more intense colors indicate stronger non-additive effects of multiple stresses: synergistic effects are shown in blue, and antagonistic effects in green. The symbol ⊗ denotes significance at *P* < 0.05 relative to full additivity.

The heatmap in **Figure 8** highlights that the combined application of the stress factors did not always produce additive effects. In particular, in some cases the interactions between stressors appeared to be stronger than expected [i.e., in ^15^N fertilizer recovery for SS(T2), and in WUE for SS(T2) and Δ^13^C for SS(T1)], whereas in other cases they were weaker than expected [i.e., in root biomass for SS(T2)].

## 4 Discussion

The system employed to control thermal heating enabled temperature increases to remain below the predetermined thresholds. Specifically, during the growth period, average temperature increments of 1.1 °C were recorded in the T1 treatment (corresponding to an efficiency of 73%), whereas, when the threshold was set at 4.0 °C (T2), the mean temperature increase observed was 1.9 °C (48% efficiency). Comparable temperature increases have been reported in other studies using IR lamps to warm ecosystem temperatures (Marchand et al., 2004; Sullivan et al., 2008). The power output of the lamps was evidently insufficient to achieve the targeted thermal increase. Notably, temperature variations induced by the lamps exhibited substantial diurnal fluctuations, with the system proving less effective during daytime hours and under higher ambient temperatures, consistent with previous observations reported by Wall et al., 2011. and Johnson et al (Johnson et al., 2013). Such an effect may be attributed to an increase in latent heat loss resulting from enhanced heating, as well as to wind variability, which, according to Sullivan et al. (2008), accounts for over 50% of the variation in IR lamp effectiveness. Overall, the system employed to control thermal heating proved to be effective because, although it did not allow the programmed temperature deltas to be maintained for the entire duration of the experiment, it nevertheless produced significant temperature increases relative to ambient conditions. Furthermore, although the asymmetry of warming between daytime and nighttime may introduce an additional layer of complexity in interpreting plant responses—for instance, by affecting processes related to plant carbon metabolism to different extents (Sadok and Jagadish, 2020)—it should be noted that climate models consistently indicate that future temperature increases will also vary by time of day, with warming often projected to be greater at night than during the daytime (Donat and Alexander, 2012). Therefore, in contrast to experimental designs based on fixed, non-fluctuating temperature increments, the diurnal asymmetry observed in our experimental system, although not intentionally imposed, mirrors in some way a realistic feature of projected climate change scenarios.

This study provides robust empirical evidence that the simultaneous occurrence of warming and either drought or salinity markedly constrains maize growth and alters nutrient dynamics, sometimes in non-additive ways. In particular, the data indicate that elevated temperatures substantially exacerbate limitations in plant N uptake, especially under water stress conditions and, most notably, in the presence of salinity stress. These findings corroborate and expand upon previous research on multifactorial stress combinations (MFSC), highlighting that the interaction among abiotic stressors imposes substantially greater physiological strain on crops than each stressor acting independently (Zandalinas, 2021; Peláez‐Vico et al., 2024).

Salinity emerged as the most severe individual stressor, causing substantial reductions in shoot and root biomass, water use, and N uptake. These effects are consistent with previous findings indicating that salinity disrupts osmotic regulation and ion homeostasis, thereby constraining water absorption and nutrient acquisition (Munns and Tester, 2008). In contrast, drought induced a moderate decline in shoot biomass but stimulated root growth, reflecting a compensatory strategy commonly observed under water-limited conditions (Comas et al., 2013). Warming alone resulted in relatively minor reductions. Recent field studies conducted in Brazil on forage species have shown that a 2 °C increase (similar to that applied in the present study under the T2 treatment) can potentially promote plant growth, nutrient use efficiency, and enhance biological N_2_ fixation, but only if an optimal irrigation regime is maintained (Barreto et al., 2020; Olivera-Viciedo et al., 2021; Santos Júnior et al., 2025). On the other hand, it should be noted that the temperature increases observed in our study, although modest (slightly below 2 °C on average in T2), occurred under conditions where the mean ambient temperature was well above the species’ upper threshold for the vegetative period, which is about 30 °C (Stewart et al., 1998). Consequently, this realized temperature increase likely intensified the stress to which the plants were already subjected.

Furthermore, warming amplified the detrimental effects of both water and salt stress, particularly by decreasing WUE and total biomass production. These negative effects intensified with increasing warming (i.e., from T1 to T2), suggesting a dose-dependent response to thermal stress. This result is especially concerning given that the temperature increases effectively attained under the T2 treatments were substantially lower than those projected under the most severe climate change scenarios. Consequently, it is reasonable to infer that, had the targeted temperature increase in T2 (+4 °C above ambient temperature) been fully achieved, the observed reductions would likely have been even more pronounced.

One of the most critical consequences of rising temperatures, in our view, relates to the proportional increase in water consumption accompanying temperature elevation. Consistent with previous findings (Lehmann et al., 2013; Kiani Ghalehsard et al., 2021), rising temperatures are expected to increase agricultural water demand because of enhanced evapotranspiration driven by higher saturation vapor pressure. This leads to relatively drier air and greater drought stress when soil water supply does not increase accordingly, thereby exerting additional pressure on already limited water resources (Lobell et al., 2013). Furthermore, climate change is projected to further reduce water availability, posing a serious constraint on food production across many regions worldwide (Liu et al., 2022a).

The interaction effects were particularly pronounced. The combined application of water stress and warming [WS(T2)] resulted in a greater reduction in shoot biomass (–12%) than either stress alone, likely due to impaired evaporative cooling caused by reduced transpiration and the increased metabolic demand associated with elevated temperatures. Many crops tend to increase stomatal conductance under heat stress to enhance transpirational cooling and regulate leaf temperature (Sadok et al., 2021). However, when drought conditions prevail, stomatal conductance is restricted to minimize water loss (Li Y. et al., 2017a). The dominance of the drought signal over the heat signal, as observed in maize (Hussain et al., 2019), results in sustained stomatal closure and elevated leaf temperatures. Such overheating can irreversibly impair photosynthetic activity and other metabolic processes, ultimately leading to notable yield reductions. These findings highlight the complex interplay between heat and drought stress responses and emphasize the need to consider their combined effects when assessing crop resilience under future climate scenarios.

Similarly, WUE declined markedly under combined stress, showing a 24% reduction in the SS(T2) treatment, which indicates a severe impairment in the plants’ capacity to convert water into biomass.

Interestingly, root biomass exhibited an unexpected increase (+23%) under the combined salinity and high-temperature treatment [SS(T2)]. The available data do not allow us to formulate hypotheses regarding the mechanisms underlying this unexpected response, particularly given that it contrasts with the pattern observed under SS(T1). Notably, it is unlikely that this unexpected response was influenced by root confinement, as root growth across all SS treatments remained consistently lower than in the control, indicating that pot volume was not a limiting factor. Altogether, these findings highlight the need for further investigation into the molecular and physiological mechanisms driving this behavior.

Nitrogen dynamics exhibited a complex pattern. Salinity stress reduced N uptake and ^15^N fertilizer recovery, with elevated temperatures further exacerbating these declines. Other studies have highlighted that heat stress can negatively affect N metabolism by interfering with the expression of numerous genes involved in N assimilation (Li C. et al., 2017b). Interestingly, the salinity-induced reductions in N uptake and ^15^N fertilizer recovery were less pronounced than those observed for plant biomass accumulation, which likely accounts for the consistent and significant decrease in NUE under SS conditions. Nevertheless, NUE remained relatively stable across temperature treatments within each irrigation condition, suggesting that once N was absorbed, the plants’ capacity to assimilate it into biomass was not substantially affected by temperature. This apparent decoupling between N uptake and utilization efficiencies underscores the distinct physiological constraints imposed by combined abiotic stresses.

Overall, these results emphasize the multifaceted impacts of concurrent warming and drought or salinity stress on crop performance. They reinforce the MFSC framework and highlight the urgent need to develop maize cultivars with enhanced tolerance to multiple simultaneous stressors. Furthermore, although the present study was conducted under pot conditions and any extrapolation of the results to real field contexts must therefore be made with caution, the findings underline the importance of implementing agronomic strategies aimed at mitigating stress impacts, such as precision irrigation, soil amendments, besides the use of stress-tolerant genotypes (Shahzad et al., 2021; Palmgren and Shabala, 2024). This will be crucial for sustaining productivity especially in climate-vulnerable regions such as the Mediterranean region which continues to experience escalating environmental pressures driven by climate change (FAO, 2021; Intergovernmental Panel on Climate Change (Ipcc), 2023). Moreover, the present results indicate that the detrimental effects of combined stresses on plant performance became increasingly severe as temperature rose. Accordingly, given that the actual temperature increments achieved were considerably lower than those projected under the most extreme climate change scenarios for the Mediterranean region, it can be inferred that fully attaining such projected warming levels would likely lead to even greater negative impacts.

Future research should expand these perspectives, for example by targeting the reproductive stages of maize and exploring the potential contribution of soil–plant–microbe interactions in alleviating abiotic stresses and enhancing physiological resilience and yield potential. Finally, although the present pot experiment provides valuable insights into maize responses to multiple simultaneous climate change-related stressors, the inherent constraints of pot-based studies necessitate field validation, as widely recognized in the literature (Mittler, 2006; Suzuki et al., 2014) and addressed in some studies combining controlled and field experiments in maize (Liu et al., 2022b) and other crops (Chilakala et al., 2022; Kolyva et al., 2025).

## Data Availability

The raw data supporting the conclusions of this article will be made available by the authors, without undue reservation.
