# Supplementary material for: Warming enhances the detrimental impacts of drought and salinity on maize growth, water-use efficiency, and nitrogen recovery
Source: Front Plant Sci. 2026 May 1;17:1764828. doi: 10.3389/fpls.2026.1764828 (PMC13176275; doi:10.3389/fpls.2026.1764828)
Supplement: Supplementary file 1 [file Supplementaryfile1.docx]

**Supplementary material**

*Canonical Discriminant Analysis (CDA)*

To assess overall treatment differentiation across multiple variables, we conducted a Canonical Discriminant Analysis (CDA) on the nine measured response variables. The analysis was implemented using the “candisc” package (Friendly and Fox, 2025). The percentage of variance explained by each canonical axis was calculated from eigenvalues, and variable contributions were assessed through canonical structure coefficients (loadings). Treatment centroids with standard errors were plotted in the space defined by the first two canonical variates to visualize multivariate treatment separation.

Pairwise Mahalanobis D² distances between all treatment combinations were calculated using the pooled within-group covariance matrix. Statistical significance of each pairwise distance was assessed by converting D² values to χ² statistics with degrees of freedom equal to the number of variables (df = 9) and testing against the χ² distribution (α = 0.05).

**Reference**

Friendly, M., and Fox, J. (2025). candisc: Visualizing Generalized Canonical Discriminant and Canonical Correlation Analysis. Available at: https://cran.r-project.org/web/packages/candisc/index.html (Accessed November 10, 2025).

**Table S1.** Analysis of variance (ANOVA) of the nine traits measured: *P* values of temperature, water regime and their interactions.

**Table S2.** Pairwise Mahalanobis D² distances and relative *P* values between all treatment combinations [WW: control, i.e., well-watered condition under ambient temperature; T1 and T2: scheduled temperatures of +1.5 and +4.0 °C above ambient temperature; WS: water stress; WS(T1): water stress + T1; WS(T2): water stress + T2; SS: salt stress; SS(T1): salt stress + T1; SS(T2): salt stress + T2].

**Figure S1.** Boxplots showing variation around the median for Δ^13^C for each treatment [WW: control, i.e., well-watered condition under ambient temperature; T1 and T2: scheduled temperatures of +1.5 and +4.0 °C above ambient temperature; WS: water stress; WS(T1): water stress + T1; WS(T2): water stress + T2; SS: salt stress; SS(T1): salt stress + T1; SS(T2): salt stress + T2]. The grey dashed line was drawn at the mean value of the control treatment (WW). The Δ-mean and the confidence intervals (in parentheses) for selected treatment contrasts relative to the relative control treatment [(WW *vs.* T1, T2, WS, and SS; WS *vs.* WS(T1) and WS(T2); and SS *vs.* SS(T1) and SS(T2)] are also reported.
